# Supplementary material for: An interpretable ML model to characterize patient-specific HLA-I antigen presentation
Source: bioRxiv. 2023 Mar 13:2023.03.12.532264. Preprint. [Version 1] doi: 10.1101/2023.03.12.532264 (PMC10054957; doi:10.1101/2023.03.12.532264)
Supplement: 1 [file NIHPP2023.03.12.532264V1-supplement-1.pdf]

Supplementary Materials

Supplementary Note 1: Comparison of metrics

AUROC and precision at certain recall are both widely used metrics. However, the latter is more informative when the dataset is highly unbalanced, and the goal is to find a few positive examples. The definition of the three (as TPR is recall) are as follows.

$$TPR = Recall = \frac{TP}{TP + FN};\ FPR = \frac{FP}{FP + TN};\ Precision = \frac{TP}{TP + FP}$$

A relationship among these values can be deduced when the ratio of positive and negative examples are known.

$$Precision = \frac{TPR}{\frac{p(-)}{p(+)}FPR + TPR}$$

TPR (recall) and precision are more relevant to our task—select a few peptides that are likely to be binders. Here, recall indicates how many true binders we can recover, and precision tells how many decoys we have to include as a cost. FPR, on the other hand, tells the ratio of false positives and the number of decoys. It is not of immediate interest and can be misleading because a very small portion of many decoys can be disastrous. For example, when FPR=5% and TPR=90%, if $p(-){:}\,p(+) = 100{:}\,1$, the precision will only be 15%, not 100% - 5% = 95%. At this accuracy, a cancer vaccine would be technically impractical and financially prohibitive. This again indicates that a very large portion (FPR = 0.05~1.0) of the ROC has no practical value.

Using our result as an example, on HLA-A3001 (Supplementary Figure 3), epiNB achieves higher accuracy than HLAthena in almost all practically useful cases (precision > 0.2 and recall < 0.9). However, the AUROC of HLAthena (0.9830) is slightly higher than epiNB (0.9810). Since TPR is another name for recall, we can find the FPR at 0.9% FPR, which is clearly smaller than 0.1. This means that the small (and arguably useless for its low precision) portion (recall > 0.9) in the precision-recall curve actually decides > 90% of the AUROC.

In conclusion, we believe that precision at 40% recall is a more useful metric than AUROC for cancer vaccine design.

Supplementary Table 1: Thresholds for EpiNB at 99:1 Negative-Positive ratio

Precision (median on all IEDB datasets)	Threshold for log odds
90%	29.0
80%	23.0
70%	19.0
60%	15.0
50%	11.5
40%	7.5
30%	3.0
20%	-3.0
10%	-12.0

*The threshold may not be reliable for precision > 50%. Performance are significantly different on different alleles.
